# CABots and Other Neural Agents

**DOI:** 10.3389/fnbot.2018.00079

**Published:** 2018-11-26

**Authors:** Christian Huyck, Ian Mitchell

**Affiliations:** Department of Computer Science, Middlesex University London, United Kingdom

**Keywords:** cell assembly, embodied cognition, neurocognitive model, turing test, closed-loop agents

## Abstract

The best way to develop a Turing test passing AI is to follow the human model: an embodied agent that functions over a wide range of domains, is a human cognitive model, follows human neural functioning and learns. These properties will endow the agent with the deep semantics required to pass the test. An embodied agent functioning over a wide range of domains is needed to be exposed to and learn the semantics of those domains. Following human cognitive and neural functioning simplifies the search for sufficiently sophisticated mechanisms by reusing mechanisms that are already known to be sufficient. This is a difficult task, but initial steps have been taken, including the development of CABots, neural agents embodied in virtual environments. Several different CABots run in response to natural language commands, performing a cognitive mapping task. These initial agents are quite some distance from passing the test, and to develop an agent that passes will require broad collaboration. Several next steps are proposed, and these could be integrated using, for instance, the Platforms from the Human Brain Project as a foundation for this collaboration.

## 1. Introduction

A good, perhaps the best, way to get an AI that passes the Turing test (Turing, 1950) is to closely follow the human model. This does leave a wide range of options, but one path is to build systems that are situated in environments (Brooks, 1991), function over a wide range of domains, are sound cognitive models, and follow human neural functioning and learning. Others (e.g., Hassabis et al., 2017) have made similar arguments.

It is relatively easy to argue that learning, functioning over a wide range of domains, and being situated in environments are all necessary for a system to pass the Turing test. However, the benefit of following the human models is far from straightforward, particularly as knowledge of those models is far from complete. Nonetheless, there are significant islands of evidence and confidence in psychology, linguistics, neuroscience, and related fields. For example, the Nobel Prize winning Thinking Fast and Slow (Kahneman, 2011) in psychology, The Foundations of Language (Jackendoff, 2002) in linguistics, and the Nobel Prize winning work on the brain's positioning system (e.g., Morris et al., 1982) in neuroscience. So, our research group has spent roughly the past decade building agents^1^, based on simulated and emulated neurons^2^, that function in physical and virtual environments. The emergent psychological function of these agents is measured by their behavior as neuro-cognitive models.

It seems very unlikely that our group, working alone, will build a Turing test passing agent. Indeed, it seems unlikely a Turing test passing agent of any form will be developed in the next decade. Consequently, this work is a part of a larger research effort that includes other agents and is open to all researchers and developers. In particular, our focus has switched to the Human Brain Project (HBP). The HBP has a standard suite of modeling tools, and hardware resources, including high performance computers, neuromorphic hardware and virtual environments, that are accessible, interactively via the Internet, to the wider scientific community. These provide practical platforms for developing agents in artificial neurons, and a place to develop a community of neural agent developers and researchers.

The discussion of the proposed development of Turing test passing agents start with section 2, which discusses the Turing test and what is needed to pass it. Section 3 discusses a range of agents, called CABots, developed by our group. The agents range from simple open-loop agents (see section 3.1), through closed-loop ones that take simple commands from a user (see section 3.2), to agents that have long-term memory (see sections 3.3 and 3.4). Our group is not alone in developing agents based in neurons, and section 4 describes some agents developed by others.

While several cognitive models have been developed in the CABots, these agents are relatively simple, domain specific cognitive models. For instance, while the agents learn in the cognitive sense, their learning of spatial cognitive maps and rules (Belavkin and Huyck, 2010) is extremely simple.

As the goal of this work, to develop Turing test passing systems, is distant, some possible next steps are presented in section 5. Neural systems can be developed with topologies that are clearly not biologically plausible; components based on such topologies can still be useful as they provide scaffolding to build future, more powerful systems that will have plausible topologies. Some sample next steps that the authors are particularly interested in exploring include semantic net like memories, and continuously valued Cell Assemblies to, among other things, support development of spiking neural models that behave like 1980s connectionist models (e.g., Rumelhart and McClelland, 1982; Maes, 1989). Other suggestions include spatial memory, improved vision, and episodic memory.

## 2. How to pass the turing test

A great deal has been written about the Turing test (Turing, 1950), competitions based on it are run regularly^3^, and it is the standardly agreed test for Artificial Intelligence. A brief paraphrased summary is that there is a human judge in one room, an unseen computer in a second room, and a second unseen human in a third room. The judge communicates with the other two via text. If the judge cannot decide who is the human and who is the computer, the computer has passed the test and is considered intelligent. The spirit of the test includes an open ended conversation, and two reasonable humans. It could be usefully extended to multiple tests to allow statistical significance, with multiple judges and with none choosing at better than chance. The test is not a trick (Harnad, 1992); though claimed results of passing or almost passing have been made, no artificial system has come even close.

For an AI system to pass the Turing test, it must function in a wide range of domains; after all, the conversation is open ended. The judge will be able to discuss any domain. The system does not need to know about every domain; humans do not. It does need to know about many domains, because humans do.

One easy way to know about many domains is to learn about them. Moreover, the system will need to learn during the conversation. Also, from a software development standpoint, if there is a lot of knowledge to encode, it is easier for the system to learn it than for developers to encode it.

Human learning is much studied, complex, and still poorly understood. Humans learn semantic knowledge (Quillian, 1967), episodic knowledge (Tulving, 1984), spatial knowledge (Morris et al., 1982), and other types of knowledge. There is short-term memory, long-term memory, and a range of durations in between (James, 1892). Human memory is sophisticated enough to learn the semantics of a range of domains. This deep semantic knowledge enables humans to understand domains in ways that machines are not currently capable.

For example, one technique for current systems that attempt Turing test like tasks is to get information dynamically from the Internet (Ferrucci et al., 2013). This is a shallow semantics approach. In general, it is not going to be able to answer questions like:

Are crocodiles good at running the steeplechase? (Levesque, 2014).

because the answer is not already on the Internet. To answer a question like this, deep semantics are needed. The point made by Levesque (2014) is that a Turing test judge can ask arbitrary questions like this. No system can find the answer from the Internet, or from caching away answers. The system needs an understanding of how crocodiles move, and what is required to run a steeplechase. It needs the ability to reason about these things.

Many, maybe all, of the domains people learn are grounded in the physical world. Humans typically learn to walk, use tools, eat, and to build structures. We learn how animals move, people dress, and voices sound^4^. Consequently, it is all but essential that the system exists in a rich environment, so that it can learn the deep semantics associated with the environment (Brooks, 1991). This includes not just what the objects are, but what they can be used for (affordances) (Gibson, 1986); it includes how the environment changes, and how the agent can change the environment; and it includes mechanisms for internally simulating these changes. There are many issues around embodiment (Wilson, 2002), but it is clear that a Turing test passing agent will include time pressured cognition, while still being able to abstract from the environment. It will be able to make and execute plans. Similarly, the concept of agent can be defined as an individual, that acts upon an environment for its own benefit (Barandiaran et al., 2009).

Moreover, it is clear from a psychological perspective that a great deal of, and probably most, learning is unsupervised (Reber, 1989). Fortunately, from a neural perspective, biological evidence points toward Hebbian rules, which are unsupervised. There is evidence for reinforcement learning using the dopamine system (e.g., Holroyd and Coles, 2002), but this still has an unsupervised component.

While it has been shown that systems implemented in neurons can process symbols, for Turing test passing intelligence, these symbols need to reflect rich associations with complex environments, often referred to as grounding (Harnad, 1990). In humans, symbols, in particular words, have deep links to underlying meaning. This meaning has been learned through extensive interaction with the environment (Taddeo and Floridi, 2005). The word *crocodile* is more than just a symbol. In a typical human, the word can bring up links to an immense store of knowledge about *teeth, handbags*, how they move, how they hunt, and much more. Thus, symbol processing in humans is usually much more than simple syntactic processing. Reading a sentence allows people to create a rich semantic representation, which can be stored.

It is less commonly argued that a Turing test passing system needs the performance of open domain cognitive models. A system could have cognition but cognition that does not approximate human cognitive behavior. Perhaps it is beyond the spirit of the Turing test, but a judge could try, for example, a Stroop test (Stroop, 1935), which measures interference. Nonetheless, if the system has the performance of a good cognitive model, it will only make it easier to pass the Turing test. Moreover, these models support a range of cognitive activities. If it does not perform like a good cognitive model, it will not duplicate human behavior well.

It is quite difficult to argue that a Turing test passing system must follow the human neural model. Indeed, the authors feel that eventually non-neural systems will pass the test. However, there are a vast number of challenges to meet to pass the test, and these may be most easily met by following a system that already can pass the test, human neurons.

Developing a full-fledged Turing test passing agent is unlikely to be entirely straightforward. Even the direct approach of copying human neural topology is not currently viable; among other issues, the topology is unknown, the basic neural models are not clear, and the neural dynamics are unclear.

So, if an agent, running in artificial neurons, learns and acts cognitively like humans, it can drive behavior in a complex environment. With sensors and effectors, it may become an agent that can learn the deep semantics of its environment, gaining a rich understanding of the objects an actions permissible in the environment, and mechanisms for predicting how the world will change on its own and in response to actions. This section and indeed this paper argues that, if the agent performs well enough in that environment, and the environment is sufficiently sophisticated, the agent will be able to pass the Turing test. Of course, this is just argument. The real proof will be the Turing test passing agent. How can such a system be developed?

## 3. The CABots

It is easy enough to propose that the best way to build an AI is to make a human-like neural agent. In an effort to make this happen, over the past years, the authors and collaborators have developed several virtual agents, virtual robots, with all of the processing done in simulated neurons^5^.

In the development of our agents, our group has made some scientific and engineering decisions that should be made explicit. First, all of the processing needs to be done in simulated or emulated neurons. A wide range of neural models can be used, and indeed, a given agent might have different types of models within it. The neural models generate spikes. These are widely used models of biological neurons, and there is considerable evidence that spiking is the basis of Hebbian learning (e.g., Bi and Poo, 1998). Spiking neurons also provide more and more rapid information than rate coded neurons (Schwalger et al., 2017). Similarly, there is no hardware restriction. Second, the agents need to have different types of learning mechanisms both neurally and psychologically. At the neural level these will include short and long-term depression and potentiation. There should be a reinforcement mechanism, and learning should be unsupervised. Currently, we are assuming all neural learning is Hebbian. Third, the agents make extensive use of CAs (see below); all processing may not be done with CAs, but a great deal of it is. Fourth, it is an engineering task, and the agents need to be constructed. This means that, at least in the short-term, some degree of modularity is needed; the topology needs to be constructed from parts that can be tested independently. Different sub-topologies can be combined via synapses between neurons. Eventually, the neural network will need to learn across these boundaries. Finally, the agent needs to perform as neuro-cognitive models; this is the link to psychology. Our current topologies are not accurate models of human (or other animal) topologies, but simplifications. Neural constraints are important, but the engineering constraint of getting an agent working, at this stage, is necessarily, for practical reasons, more important.

One key concept in neuroscience is the Cell Assembly (CA) (Hebb, 1949); a CA consists of a group of neurons, and is, among other things, the widely agreed neural basis of concepts (Guzsaki, 2010). When a concept is in short-term memory, the neurons in the CA are firing at an elevated rate. The formation of the CA, so that it can fire persistently, is a long-term memory. These agents make use of CAs, so, they are Cell Assembly roBots: CABots.

These agents are embedded in virtual environments, and several simple environments have been used. The agents have also been developed using several different point neural models, including a Fatiguing Leaky Integrate and Fire (FLIF) model (Huyck and Parvizi, 2012), and conductance based, and current based exponential integrate and fire neurons with adaptation (Brette and Gerstner, 2005). Point neural models are relatively simple models that treat the neuron as an input output equation; there are numerous models that are more complex (Brette et al., 2007).

Generally, when working with a new environment or neural model, an open-loop agent is initially developed (see section 3.1); these are typically very simple. Then more advanced closed-loop agents are developed (see section 3.2). Perhaps the most sophisticated agents our group has developed used the FLIF model (see section 3.3), including several cognitive models. More recently, agents have been developed for the HBP (see section 3.4).

### 3.1. Open-loop agents: CABot1

It is relatively simple to make agents with all of the processing in simulated neurons. Section 4 describes several of these agents developed by other researchers, and this paper will start discussion with a simple agent emulated on the BrainScales (Schemmel et al., 2010) neuromorphic platform. BrainScales is analog hardware with each neuron directly implemented in hardware; it emulates neurons at 10,000 time speedup over biological time. This agent takes a command input and input from a picture of the environment. It can turn in response to the command, or if the command is *turn toward the object* it will. For example, if there is a colored object on the left of the picture, a particular neuron spikes, and if it is on the right, a different neuron spikes.

The standard middleware for the HBP for describing topologies of neurons is PyNN (Davison et al., 2008). This describes the topology, and then passes that to the backend to simulate (e.g., NEST) or emulate (BrainScales or SpiNNaker).

Note that one of the great advantages of using neural systems in general, and neuromorphic systems in particular is their innate parallelism. The processing is distributed between the neurons, and even on a serial machine, processing with neurons provides algorithmic scaffolding for parallel processing; write the program in neurons, and it is already parallel because all of the neurons function independently. On neuromorphic machines, the parallel processing is rapid, and for the overall system to be expanded, all that is needed is more neuromorphic hardware. Delivering the spikes to the appropriate neurons is one of the problems with this parallelism. Perhaps the main advantage of the SpiNNaker system (Furber et al., 2013) is the mechanism that allows all spikes to be delivered in the next millisecond.

The BrainScales agent is an open-loop agent. It senses the environment, but any changes it makes do not effect the environment. Moreover, the agent does not have any neurons that fire persistently without environmental input. A CA can fire persistently, so this agent is not a CABot.

CABot1 refers to an open-loop agent that uses CAs. Several have been developed and they can take commands from a user in natural language, view the environment, make simple plans and act depending on the context of the environment. They take advantage of a simple representation of a CA. They do not get feedback from the environment, so are unable to, for instance, explore the environment by turning around.

CAs, once activated, need to persist. A good cognitive model, of a CA, of for instance a word, would have the firing rate of neurons in the CA decay like a short-term memory, but a simple well connected topology based on a point neural model is a reasonable proxy. It can remain persistently active indefinitely. A set of neurons, for instance five, is well connected, with each neuron synapsing to the other four neurons. It is relatively simple to find parameters so that once all of the neurons fire, there will be persistent firing. That is, the first time all of the neurons fire, they will cause each other to fire again, and this will be repeated; this is called CA ignition. This is a binary CA, either on (ignited), or off (not ignited); there is no intermediate level of neural firing.

These simple CAs can be used as states in a finite state automaton, and many functions can be implemented. For instance, simple regular languages can be parsed (Hopcroft et al., 2006). This enables the users' text commands to be processed by the agent.

Similarly, CAs can be used for simple plans. The overall thinking is to follow the Maes nets (see section 3.4 and Maes, 1989), but binary CAs can be used for planning.

**Figure 1:**
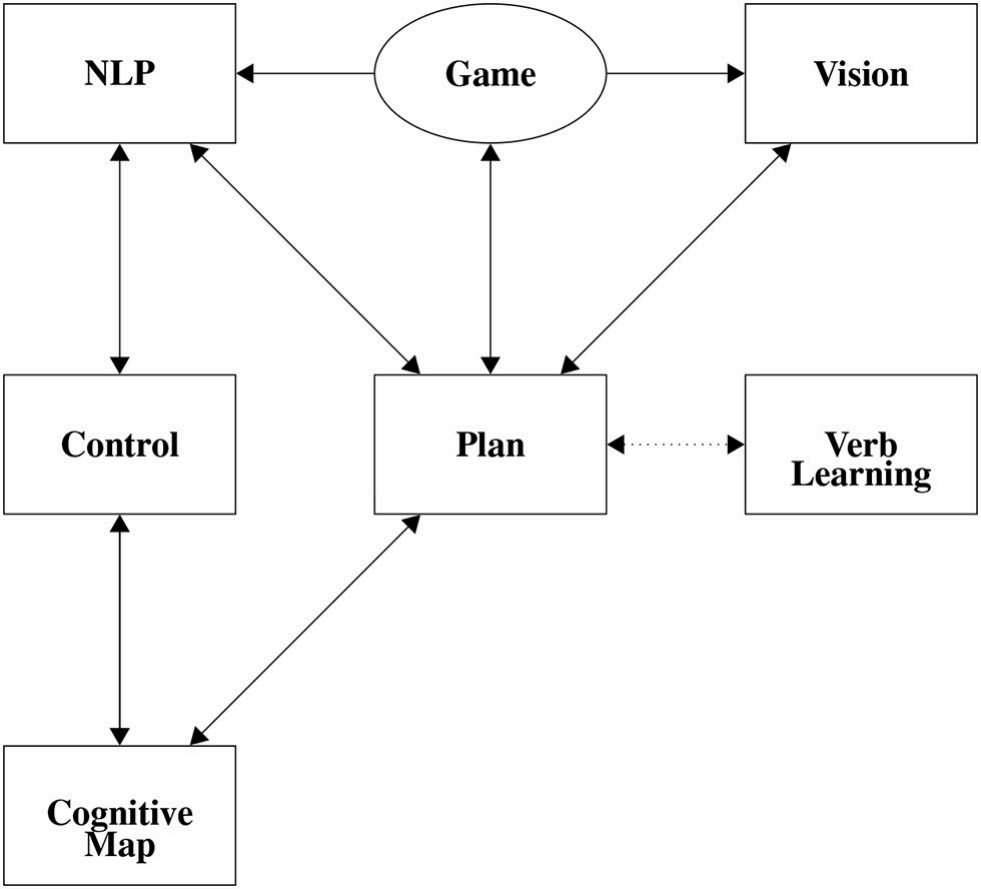
Gross Topology of CABot3. Boxes represent subystems of subnets. The oval represents the environment.

To simplify engineering and more easily understand these agents, they can be broken into subsystems. Figure 1 describes the subsystems of a more complex CABot3 agent. The CABot1 agents use an environment, Natural Language Processing (NLP), Vision, and Planning subsystems, and have most of the connections from Figure 1. However, as the CABot1s are open-loop agents, there is no connection from planning back to the environment. Individual subsystems, neurons and synapses, are built and tested in isolation. These are then combined by adding synapses between the subsystems yielding a complete agent, and the subsystems' topologies can be copied and re-used in different agents.

The visual system is probably the best understood of human neural systems, though of course understanding is far from complete. Building from early studies (Hubel and Wiesel, 1962), and making use of animals there has been steady advancement in understanding neural visual processing.

The agents our group has developed take advantage of on-off and off-on center surround processing. These come in different granularities, in our agents typically 3 × 3, 6 × 6, and 9 × 9. The visual environment is pixelated giving the visual input. This input stimulates the center surround receptors, and these in turn feed into line, edge, and angle detectors, behaving like neurons in the primary visual cortex.

There are several CABot1 agents that take commands from a user. Using the commands they set goals. These tasks are typically primitives (like *Turn left* and *Move forward*) or context sensitive (like *Turn toward the pyramid*). The visual system may fail when, for instance, the pyramid is too far away. If the visual system gives a correct interpretation of the environment, these commands are always successful, issuing the correct action as described by a particular neuron firing.

### 3.2. CABot2 and closing the loop

If the agent is in an environment, can modify the environment, sense the change, and uses the change to continue on, it is a closed-loop agent. The, CABot2, agent now becomes part of the environment, and the environment part of the agent. Fortunately, particularly in dynamic 3D environments, like many virtual environments and the real world, there is an obvious separation between the agent and the environment (Diaper and Sanger, 2005). The agent has processing, sensing, and effecting, and the environment is everything else.

Recently, a CABot2 agent was developed for the HBP's Neurorobotics Platform (NRP) (Roehrbein et al., 2016). This Platform supports virtual environments and robots driven by simulated neurons; it can be accessed over the Internet and users can develop experiments with novel virtual robots, environments and brain models.

This CABot took one of five text commands (turn left, turn right, move forward, stop, or move to the box). These commands were interpreted, neurally, by a regular grammar processor, with the result of setting a goal. One goal, move to the box, was context sensitive. This environment is a simple flat surface with a blue box on it. The robot is wheeled. There is a camera on the robot, and the results of this are sent to the visual subsystem. The visual subsystem then determines whether the box is on the left or right or directly in front, enabling the robot to move to the box when that is the goal.

There are a range of environments for agents. 3D virtual (or physical in the case of robots; see section 4.3) environments are of particular interest because of their potential richness.

Communication timing between the neurons and the environment is also important. Neurons can be readily tied to time, as they model the behavior of actual neurons by time. The environment may also be tied to time. However, the coupling of the neurons with the environment can range from loosely coupled, to tightly coupled depending on the system. Physical robots, run by neural networks, are typically tightly coupled, with input from the robot's sensors going to the neurons, and the neurons needing to respond quickly. If however, the environment does not change rapidly, the neurons may sense change in the environment, process for as long as necessary, and then respond; the agent, loosely coupled with the environment, can perform its tasks. With virtual environments, the actual time of simulation may not be the same as the simulated time. Virtual environments, can run more slowly or rapidly to correspond to simulated or emulated neural time. The latency that occurs in nature is due to time to synchronize the brain with the environment - there are many experiments that exploit this behavior, and it is inherently a closed-loop problem. Some details of synchronizing the visual subsystem with the environment to complete the closed-loop are discussed in the next two sections.

### 3.3. FLIF closed-loop agents

Perhaps the most advanced closed-loop agents that the group has developed are the CABot3s in the FLIF model (Huyck et al., 2011). The agents consist of several subsystems that can be seen in Figure 1. These were developed in our own Java FLIF simulator, and in a virtual environment using the Crystal Space games engine (Crystal Space, 2008)^6^. The environment is 3D with the agent able to move about in the environment via four primitive actions: turn left and right, and move forward and backward, all in discrete steps. The virtual environment only changes in response to the agent's movements so the two are only loosely coupled; consequently, getting information from and sending information to the environment is relatively simple. The environment consists of four rooms connected by four corridors. In each room there was a unique shape: a pyramid or stalactite that have vertical or horizontal stripes (see Figure 4).

The advancement of CABot3s over CABot2s is long-term memory. CABot2s implement short-term memory by neural firing. For instance, parsing in the NRP CABot2 makes use of persistently firing neurons to maintain memory. In the FLIF CABot3, long-term memory is formed by permanent synaptic weight change that associates a room with the object in it, so the system cannot relearn if the objects move.

The subsystems typically consisted of several subnets. A subnet consists of a set of neurons. The subsystem and subnet mechanism allow some degree of modularity for software development. These agents have the most sophisticated visual subsystem that our group has developed. In addition to subnets of neurons that performed the function of the retina, primary visual cortex, and object recognition, these visual subsystems have grating cell subnets to recognize texture. This enables the subsystem to recognize the four types of objects: vertically striped pyramids, horizontally striped pyramids, vertically striped stalactites, and horizontally striped stalactites. The NLP system refers to vertically striped objects as barred, and horizontally striped ones as striped.

These objects are used for a simple spatial cognitive mapping task. When the agent is told to explore the environment, it finds the object in the room and maps the room to that object. It then navigates through the corridor to the next room, and so on until all four rooms are mapped. This is a very simple form of long-term learning. The agent is tested by a command like, *Go to the room before the striped pyramid*. It then uses its long-term memory to retrieve that, for instance, the barred stalactite is in the room before the striped pyramid. It then traverses the environment, checking each shape, until it gets to the room with the barred stalactite, fulfilling the goal.

The NLP subsystem is quite sophisticated. It is perhaps the best neuro-cognitive model of natural language parsing (Huyck, 2009), parsing in cognitively realistic times, appropriately resolving prepositional phrase attachment ambiguity, and producing semantic output (in this case, for planning). It is currently one of the most important works in the CABot project.

This, and other CABot NLP systems, takes commands from the user, and uses them to set goals in the planning subsystem. The user types the commands into a text box in the virtual environment.

**Figure 2:**
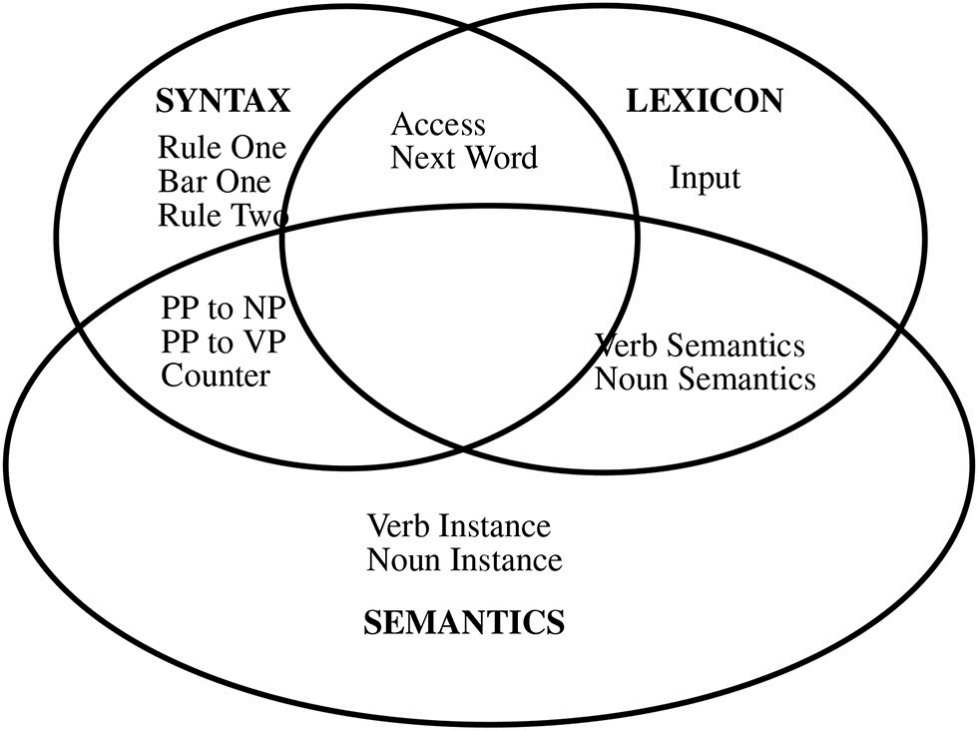
Gross Topology of the FLIF CABot3 Parser. Each box represents a subnet with similar subnets grouped together according to Jackendoff's Tripartite theory.

The system implements Jackendoff's tripartite theory (Jackendoff, 2002), illustrated in Figure 2. Subnets refer to particular sets of neurons, with all neurons in exactly one subnet. They appear in their own window in the neural system's user interface. The tripartite theory refers to three language systems, semantics, lexicon, and syntax. These systems communicate via shared sets of neurons (subnets), and Jackendoff proposes that there are other linguistic and non-linguistic systems.

Earlier versions of the system used a stack, but this did not lead to correct parse timing. So, the system uses a memory based solution, with semantic frames represented by CAs forming the basis of phrases during parsing. Binding is essential for parsing context free grammars, and binding is done by short-term potentiation (STP) in this system.

One assumption made in this work is that a concept is psychologically active, when its neurons fire at an elevated rate. As each cycle of the simulator is tied to 10 ms. of real time (Huyck and Parvizi, 2012), parsing rules are applied when their neurons are firing, and this time is readily measured. This is used to show that parsing is done in psycholinguistically realistic times. Typical neural simulations use a time step of 1 ms, 0.1 ms, or even 0.01 ms. One benefit of a 10 ms. time step is that approximately 100,000 neurons can be simulated on a standard PC in a reasonable time.

**Figure 3:**
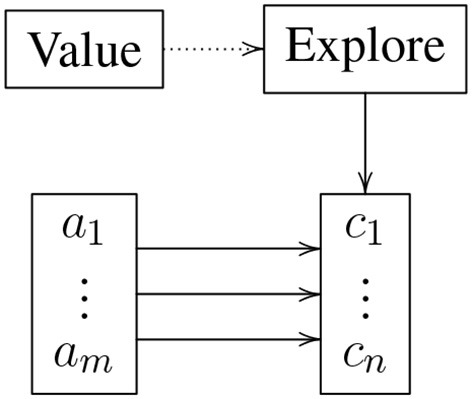
Gross topology of the reinforcement learning system. The *Value* subnet represents the reward and *Explore* supports action when there is reduced information. The *a* subnet is the collection of antecedents, and the *c* subnet the consequents.

Similarly, one variant of the agent, was a cognitive model of rule choice (Belavkin and Huyck, 2010), taking advantage of a reinforcement signal from the environment to learn the meaning, from the perspective of the agent, of centring an object. A different network, independent of the agent, used a similar topology to model a two choice task. Figure 3 describes the gross topology of this system. In the centring system, there were two antecedents: the goal center and the fact object on left, and the goal center and object on right. These came from the planning subsystem. The consequents were the action turn right and turn left, again from the planning system. Note that the theory applies to any antecedent consequent set. The system needs to learn the correct weights between the antecedents and consequents. If the weights were already learned, the correct consequent tended to be applied to the antecedent. However, the weights were initially low, so this application did not occur. Instead the neurons in the *Explore* subnet fired at an elevated rate when any antecedent was present, causing a consequent to be applied. If this led to a good result, the *Value* subnet was externally activated, leading to its neurons firing at an elevated rate, suppressing firing in the *Explore* subnet. This meant that the correct antecedent consequent pair fired, and the weights from the antecedent to the consequent were increased due to Hebbian long-term potentiation. Similarly, weights from the antecedent to incorrect consequents were reduced via Hebbian long-term depression. If, on the other hand, the incorrect consequent was selected, weights from the antecedent to the incorrect consequent were initially increased. However, the *Value* subnet never came on, so the *Explore* subnet continued to be highly active, leading to a new consequent being selected. As this process is repeated, the only attractor states are the correct antecedent consequent pairs as determined by the reinforcement signal to the *Value* subnet.

Moreover, this rule selection mechanism is not static. If the environment changes, the reinforcement mechanism in collaboration with Hebbian learning will learn the new utilities of the rules.

The use of our own neural simulator and model, and our own virtual environment has its advantages. If there is a minor problem, or a new learning rule is needed, it can readily be implemented. Unfortunately, this means that no one outside of our group has ever used these systems. While some students have been taught to use and modify the FLIF agents and the code has been made available, it seems the learning curve is too steep. Another approach is to use more widely used tools to build modules that can be used in many agents, and by many researchers.

### 3.4. HBP closed-loop agents

One of the problems with the FLIF CABot3 agents was processing time. As more neurons were used, simulating on a standard PC began to become quite slow. While the loose coupling of the virtual environment and the agent addressed time sensitivity, adding more neurons slowed the machine markedly; at one point, the Java heap could not be further expanded. Recently CABot3 agents have been developed for two of the HBP computational platforms: SpiNNaker neuromorphic hardware (Furber et al., 2013), and NEST (Gewaltig and Diesmann, 2007) simulations. One of the benefits of these platforms is speed. SpiNNaker simulates neurons in real time, typically at 1ms clock speeds. So, as the number of neurons in our simulations grow, the run time speed remains constant. NEST is readily parallelisable and can be run on high performance computers, though we run on a standard PC.

The HBP CABot3s run in a new virtual environment written in Python using the Tcl visual libraries. The agents run in both NEST and SpiNNaker using the same code; there are the same set of neurons and synapses in both version, but in some cases the synaptic weights differ. They perform the same four room cognitive mapping task as the FLIF CABot3. They no longer use texture (vertical and horizontal stripes), replacing these with color (red and blue). The corridors are now green. These colors considerably simplify visual processing. NLP no longer uses variable binding, as the STP rules standardly available in NEST and SpiNNaker cannot be readily used for this task. So, in the HBP CABot3s, parsing uses regular instead of context free grammars (Hopcroft et al., 2006).

An early version of the agent ran only on SpiNNaker, and used current based exponential integrate and fire neurons with adaptation (Brette and Gerstner, 2005). The current based version is not currently available on BrainScales, so the current version uses conductance based exponential integrate and fire neurons.

SpiNNaker, and to a lesser extent NEST, is still under development, and its underlying software is changing. Though it is becoming more stable, changes in that software required the earlier agent to be rewritten. The agents' dependency on precision of behavior of neurons was tight and implicit; any changes to the agent required complex rewriting. Consequently, the current agents have been developed with the NLP, planning and cognitive mapping subsystems making extensive use of a Finite State Automata (FSA) class. So, when the underlying neural model is changed, it is simpler to update the subsystems and agents. Moreover, new components can be added making use of FSAs. Similarly, a timer class, similar to a synfire chain (Ikegaya et al., 2004), has been developed and is used in the planning and cognitive mapping subsystems. As software developers know, software needs to be maintained, and this includes these neural components.

Natural language parsing, to set the goals, is done using binary CAs to implement FSAs. Simple plans can also be implemented using FSAs, and this is the mechanism used for simple goals like *Move forward* or *Turn toward the pyramid*. It however proved more difficult to implement more complex movement, like that needed to explore the four rooms, using binary CAs alone. Maes nets (Maes, 1989) use connectionist units that have a continuous value, and spread activation between units; these are similar to the interactive activation model (Rumelhart and McClelland, 1982). Maes nets have units for goals, modules, facts, and actions. Activation spreads between the units, and when an action unit reaches sufficient activation, it is chosen and applied. Implementing these multivalued units cannot be readily done with binary CAs. However, timers, implemented in neurons, can be used in collaboration with binary CAs to approximate this behavior. For example, facts are stimulated by the environment, however, they should only be turned on when an appropriate goal is active. If there is a pyramid in the right of the visual field, it will not alone turn on the associated fact. However, when the goal *Turn toward the pyramid* is on, it will turn on a timer that sends extra activation to the pyramid on left and pyramid on right fact CAs. In collaboration with the environment, the appropriate binary fact ignites. Moreover, multiple timers can be used for particular goals. For instance, if no fact is active even after the first timer, a second timer can be activated to perform a second round of activation of other facts. If this is unsuccessful, a default action may be taken.

The spatial cognitive mapping subsystem interacts with the planning subsystem. It learns associations between the four rooms and the four shapes in them; there are CAs for each of the rooms and for each of the shapes. All are connected via synapses that learn via a spike timed dependent plasticity rule (Bi and Poo, 1998). FSAs gate activity so only the appropriate CAs are active when the *Explore* goal is set. During this time, only one room and one shape are simultaneously active at a time, and these are associated. During the search for the goal, the appropriate goal shape is activated, which is gated via an FSA to activate the shape from the prior room (again with no more than one room and shape simultaneously activated). The goal is fulfilled when the agent sees that shape, which is when the vision system, based on the agent's camera in the virtual environment and planning system determine the shape is present.

The HBP CABot3s perform perfectly on the simple commands, (e.g., *Move forward* and *Turn left*), and compound commands (e.g., *Move left*, which turns left and then moves one step forward). This is due to the programmatic nature of these tasks. These are deterministic, so both the NEST and SpiNNaker version perform perfectly. There are some minor differences between the two systems with floating point numbers being 32 bit in NEST and 16 bit in SpiNNaker, but for these actions both agents are deterministic.

The most complex task is learning and using the spatial cognitive map. An example of this is the command *Explore*, followed by a command like *Move to the room before the room with the red stalactite*. These two commands fill the map and then test it has been correctly filled. Here the SpiNNaker agent loses its determinism, even when given these commands at the exact same time in the simulations, performance varies. The variance stems from the visual input from the environment to the board. This has been implemented by taking a bitmap of the environment from the agent's camera, pixelating it, and sending spikes to the associated visual input neurons. This leads to an irregular spike timing pattern, with input stopping while the picture is analyzed. Even when the picture is not being processed, input spikes to the board are not regular. So, input comes roughly every 30 ms with variance to between 20 and 40 ms, except when the picture is being processed, when input will cease for approximately 100 ms. This input variability requires both the visual subsystem and the planning subsystem, which gets input from the visual subsystem, to be more flexible.

The planning subsystem is responsible for the agent's temporal behavior. While pursuing some goals, it uses input from the visual subsystem to determine the agent's relative spatial location. During exploration, it sends this information to cognitive mapping, and retrieves that information when starting a *Move before* goal.

With NEST, the input is entirely regular. The actual neural time does not need to correlate with the real time, as the environment and the neurons are loosely coupled, so input comes every 30 ms. Nonetheless, the system is still complex enough that it behaves differently each time on the *Explore* command. The inputs come every 30 ms of simulated time, but variance creeps in immediately, since the camera to pixel mechanism from the environment has variance. So each action sequence is different. If input timing was regularized in SpiNNaker, this task would still be non-deterministic.

**Figure 4:**
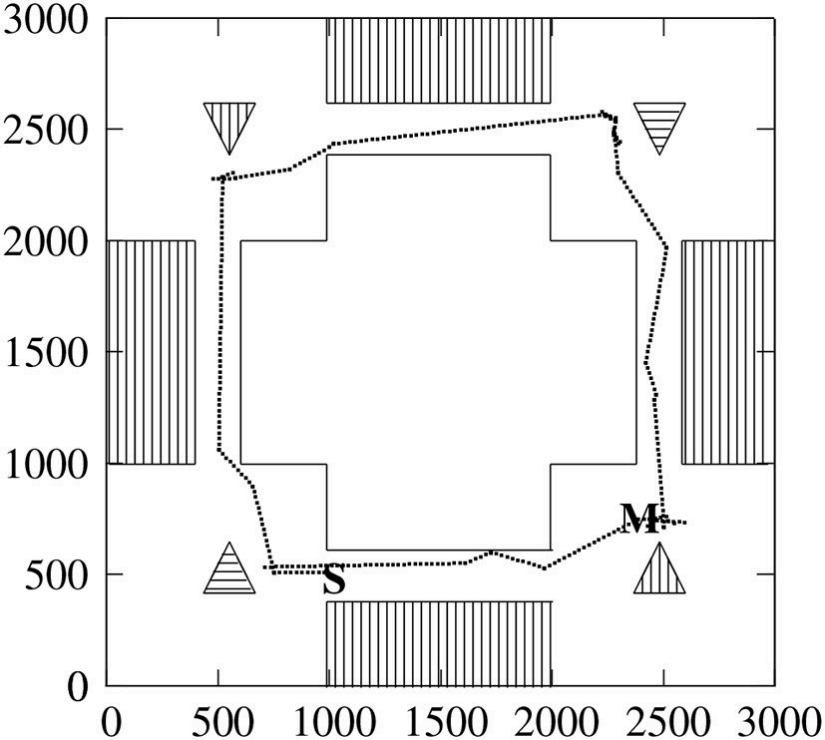
Moves of CABot3 while executing the *Explore* command followed by the *Move before the red stalactite* command. This is a top down representation of the environment. Moves are marked by dots. The agent starts at S, and the move command is executed at M. The outside of the box represents the walls as do the stripes on the inside. The blue stalactite and pyramid are represented by horizontal stripes, and the red objects by vertical stripes. The pyramids point to the top of the page, and the stalactites to the bottom. The numbered axis units are Tcl points.

Figure 4 shows one instance of the agent performing the *Explore* task, followed by the *Move before the red stalactite* task. Movements around the red stalactite and blue pyramid show the difficulty the agent has identifying the object, making several moves to identify it. After one Move command, the agent can perform others, though it can only explore once. This task is complex requiring well over one hundred primitive moves. The agent must identify the four objects, and navigate through the four narrow corridors between the rooms.

The NEST version of the agent does these two commands correctly 86/100 times, and the SpiNNaker version does it correctly 49/100 times. The measurement is done over 200 s of neural time. The task is typically completed in about 85 s, but given a longer time, the actual results will be higher. The agents do fail at these tasks. For example, one failure arises from incorrectly identifying an object, with a pyramid being substituted for a stalactite or vice-versa. An improved plan, or an improved visual subsystem will lead to better performance.

There are several reasons for the system failing. As visual input is only 20 × 20 pixels, viewing the objects at a distance does not provide the agent with enough information to distinguish a pyramid from a stalactite. The plan is also designed for speed. When exploring, the agent identifies the object, then turns right looking for the corridor, then goes to the corridor, then through it, and then back to identifying the next object. If the agent misses entering the corridor, it can go to the left or right of the entrance, and get into a state of circling the room. Similarly, while going through the corridor, it may turn around as it cannot identify the end of the corridor, it goes back to the room that it has just come from; it can recover from this.

The tasks the CABot3s must perform are sufficiently sophisticated to make them interesting. Firstly, there are many tasks that the agents must be able to perform, as set by a user. Secondly, the mapping tasks require the agent to make hundreds of moves interacting with the environment throughout. Thirdly, the corridors are difficult to find, enter, and exit. Fourthly, the visual items are difficult to distinguish from each other.

The system has an, albeit programmed, sophisticated link between cognitive mapping, vision, planning, and the language semantics. In essence, the semantics of the words are grounded in the environment; the system addresses, but does not resolve, the symbol grounding problem. This shows the agents are sophisticated, and a promising basis for future exploration.

## 4. Other neural agents

The neural agents developed by the authors' group are, of course, not the only neural agents. Several simple neural agents already exist on the HBP's NRP (see section 4.1). There are other virtual agents (see section 4.2) and there are robots (physical agents) driven by neurons (see section 4.3). This is not meant as an exhaustive review of other neural agents, but as an entry to the area.

### 4.1. Neurorobotics platform

The HBP's NRP (Roehrbein et al., 2016) has several agents that can be run interactively over the Internet^7^ using the NRP's server. Many of these agents are driven entirely by simulated neurons (using NEST). The environments, virtual robots, brain models (neural nets) and communication mechanisms can all be changed by the user. This is an excellent platform to explore and compare virtual neural agents.

One example experiment is the Husky Braitenberg experiment with virtual red detection. This agent has a simulated four wheeled robot with a camera on top of a Husky robot. This agent looks for red objects and then moves toward them. It is a virtual implementation of Braitenberg's (1984) simplest vehicle, which makes use of a simple visual color detection mechanism.

Another example is the force based joint control simulation of a hand. The virtual environment simulates the physics of a simulated robotic hand. The neurons respond to pressure, from the environment, to move the simulated finger to a specified location.

### 4.2. Virtual neural agents

There are many virtual neural agents, and many agents using connectionist systems. This section discusses some agents that use spiking neurons.

One system (Neftci et al., 2013) categorizes visual images depending on context. It uses a real-time neuromorphic architecture emulated in a CMOS VLSI system. Its task is to follow either a vertical or horizontal bar on a video screen. Depending on the context (a red or blue circle), the system must respond when the horizontal or vertical bar enters the right half of the screen. The context is provided by an FSA, and the neural system takes advantage of soft winner-takes-all networks.

Another system (Potjans et al., 2009), based on spiking neurons, learns using temporal difference and reinforcement learning, both implemented in biologically plausible learning rules. The task is to move to a reward position on a grid.

One group has developed a broad set of linked subsystems from spiking neurons (Eliasmith et al., 2012), with a wide range of functionality including vision, motion, language processing and some learning. This makes extensive use of vectors implemented by spiking neurons.

### 4.3. Robots

There is a rich body of literature on the use of neurons to drive robots. One early neural robot uses spike response neurons, a simple vision system, and a set feed forward topology (Floreano and Mattiussi, 2001). An evolutionary algorithm is used to set whether particular synapses exist and if so if they are inhibitory or excitatory. The fitness function optimizes for a robot that travels as fast as possible without hitting the walls in its environment. This is a stimulus response agent similar to several of the virtual agents described above.

Another robot parses commands and uses simple plans and vision to turn toward an object in a particular color (Fay et al., 2005). Though specialized vision algorithms are used, this system makes use of CAs and FSAs, and so, in the terminology of this paper, is a CABot2.

There is a particularly rich area of research in robot control, and learned robot control (e.g., Dean et al., 2009). For example, one system learns how to control a robot arm using spiking neurons and synapse adaptation rules, though these are based on error feedback (Carrillo et al., 2008).

## 5. Extensions

While this paper describes neural agents, a key point is that, if developed correctly, neural agents and their components can be combined, and that they can be compared. The agents described in section 3.4 and their components can be reused, improved, extended and evaluated.

The existing subsystems can be modified to perform in other environments and other tasks, such as finding an object in a maze. Existing neural subsystems can be replaced allowing comparison and improvement; for example, the spatial mapping and vision subsystems can be replaced. New modules can be added; for example, the addition of a natural language generation subsystem could make a conversational agent, and an episodic memory would support agents that persisted longer benefiting from those memories.

New subsystems can be integrated with the agents. The authors are beginning to work on a semantic memory subsystem. CAs will emerge from input, and relationships between them will be learned. This semantic net will be both a long and a short-term memory supporting several simultaneously active nodes based on input; part of the evaluation will include a neuro-cognitive model, which will duplicate priming data and perform a Stroop test (Stroop, 1935). Other example subsystems include episodic memory, spatial reasoning, motion, foveation, and emotion.

Components of the systems described in section 4, and other systems, could be included in the suite of components. Unfortunately, it is often difficult to unbundle full neural systems, but as they are already connected via synapses, there is a mechanism. This will require some development effort, but there is no reason not to start work on integration. The NRP is one platform that could be used to support integration. Developing an agent, a virtual environment, or a component can be quite complex, and combining them can be similarly complex. Developing software engineering support for these tasks would be valuable. One form of support would be benchmarks to facilitate comparison. For example, others could use the four room task to see if another neural agent can perform better.

New abstract data types, implemented in neurons, can be added, supporting the development of new subsystems and agents. The FSA and timer are already supported, but new types like soft winner-takes-all nets can be added. The authors are working on continuously valued CAs, which unlike our current binary CAs, will have a range of activity that gradually changes depending on input, and over short times. One version would be roughly equivalent to the Interactive Activation Theory (Rumelhart and McClelland, 1982) and could be used for a wide range of cognitive systems and cognitive models.

Another way forward would be to build neural perceptual symbol system simulators (Barsalou, 1999). A system would both recognize percepts, and produce them in simulation. Beyond that, a neural system that learned new simulators would be a powerful step toward agents with deep semantics.

There is also scope for advancement in improved neural models, improved topologies, and improved learning mechanisms. Here, improved means that they perform their tasks better, but also that they more accurately reflect biology. For example, the CABots described above have used point neural models. Perhaps more sophisticated models are needed, but it is not currently understood if that is the case or why. The existing, less plausible, systems can provide a scaffolding for more sophisticated and plausible future systems. Proposals do exist for more accurate and computationally viable topologies (e.g., Granger, 2006). These are obvious extensions. Moreover, these computational models can help in understanding the actual biological systems. Moreover, there is a vast range of research in, for example, power law scaling (Tinker and Velazquez, 2014) and the balance between excitatory inhibitory activity (VanVreeswijk and Sompolinsky, 1996). All of this work can be explored and integrated into neural agents.

The main advantage of neural systems is that they can learn. The CABot3 agents learn, and spiking nets can be used for a wide range of machine learning tasks (Ghosh-Dastidar and Adeli, 2009). However, agents have the ability to exist practically indefinitely, and the scope for learning is immense. The neural agents described above, in both sections 3 and 4, have been, in essence, programmed to behave. Future agents need much more learning.

Perhaps the most important task to extend these neural systems is to explore learning mechanisms that can learn over days and longer. This includes learning across subsystem boundaries. It also includes learning from the environment, as opposed to typical machine learning systems that learn a single task from refined input. Imagine a system that learned a perceptual symbol system and its associated simulators. Working in a relatively small domain, it could learn the deep semantics of that domain. As others have suggested (Gomila and Muller, 2012), the system could then learn other simple domains, possibly benefiting from its knowledge of earlier domains.

If the domain included crocodiles and steeplechases, the agent could learn those deep semantics, and answer the unanticipated question “Are crocodiles good at running the steeplechase?” As the agent learned the deep semantics of broader domains, and more domains, it would be able to answer more questions. Eventually, the authors propose, such an agent would be able to pass the Turing test.

Conversational systems aid in this ability to learn. Via the conversation, the agent can learn from a person. Moreover, by developing conversational agents, understanding of social cognition, situated cognition, and dynamic communication may be furthered.

However, to get to such agents, we need to move from programmed systems using FSAs or relatively small dimensional vectors, to more biologically plausible systems, like CA based systems where the CAs are learned, behave more robustly, and behave more realistically psychologically. If these agents functioned in complex domains, they could learn from them.

## 6. Conclusion

The scientific community is quite some distance from understanding how cognition emerges from neural behavior. An excellent way to develop this understanding is to build artificial neural systems that produce similar cognition. Systems that also produce similar neural behavior are even better.

This paper has summarized several neural cognitive agents situated in environments. These produce a range of behaviors from simple actions, to complex goal directed behavior, and perform as neuro-cognitive models. These have been developed in components so that new components can be added, existing components can be modified, and new agents can be constructed from these components.

These agents and components will provide support for further exploration of neural cognitive agents, both in the form of running systems, and with links to neuro-cognitive research. Others may make their neural systems available and usable for comparison and reuse. Reuse of systems is just good engineering, and rerunning of experiments is just good science. None the less, focused efforts, beyond the scope of even the HBP, could lead to more rapid advancement. This will require a great deal of effort and expense. There is a vast distance from these agents to the goal of Turing test passing agents, but this paper has also provided possible next steps on that path.

It is clear that the existing CABots are not close to passing the Turing test. The authors instead argue that pursuing the human model (embodied agents, based on human neural functioning, that learn, function in a wide range of domains, and are cognitive models) is the best route to developing such a system. There are a vast number of problems to overcome before such a system is developed including basics of sensing, action, and memory, but also resolving classic problems like symbol grounding and the frame problem (Dennett, 1984). These problems have all been resolved by human brains and bodies. Scientists may not know how they have all been solved, but the working model provides answers to be discovered.

While developing neural cognitive agents is a difficult task, there is an added benefit that systems that can produce cognitive behavior will be useful in their own right. A system that can learn the deep semantics of a new, but restricted, domain will be an excellent tool to work in that domain.

The neural agent approach is not only the best way to achieve the lofty and distant goal of passing the Turing test; it is an excellent way to improve our understanding of neural behavior and psychological behavior. It is also an excellent way to build more sophisticated AI systems that are tools for use in real environments.

## Author contributions

CH has done most of the development, writing, and research. IM has done a considerable amount of development on the new planning component, some writing on the paper, and has been in many discussions.

### Conflict of interest statement

The authors declare that the research was conducted in the absence of any commercial or financial relationships that could be construed as a potential conflict of interest.

## Footnotes

^1^An agent is something that perceives its environment and acts on it.

^2^Artificial neurons, in contrast with biological neurons, are simulated on standard hardware using simulators like NEST and are emulated on neuromorphic hardware.

^3^For example, the Loebner prize runs annually (see http://www.aisb.org.uk/events/loebner-prize).

^4^Even extremely physically impaired people, e.g., Helen Keller, a deaf blind person, still existed in an environment and interacted with it.

^5^The code can be found at http://www.cwa.mdx.ac.uk/chris/cabotsPaper/cabotsCode.html

^6^http://www.crystalspace3d.org/main/main_page

^7^https://neurorobotics.net/
